# Is acne in adolescence associated with prostate cancer risk? Evidence from a meta-analysis

**DOI:** 10.1371/journal.pone.0206249

**Published:** 2018-11-07

**Authors:** Xian Zhang, Yi Lin, Xiaoning Xie, Meiya Shen, Guoping Huang, Yunmei Yang

**Affiliations:** 1 Department of Geriatrics, First Affiliated Hospital, School of Medicine, Zhejiang University, Hangzhou, China; 2 Department of Oral and Maxillofacial Surgery, First Affiliated Hospital, School of Medicine, Zhejiang University, Hangzhou, China; The Wistar Institute, UNITED STATES

## Abstract

**Introduction:**

Previous studies regarding the relationship between acne and prostate cancer risk have reported inconsistent results. We performed the present meta-analysis of observational studies to summarize the evidence on this association.

**Methods:**

A comprehensive literature search up to March 2018 was performed in PubMed, Scopus, Web of Science, and Chinese National Knowledge Infrastructure (CNKI) databases. Summary odds ratios (ORs) with 95% confidence intervals (CIs) were estimated with a random effects model. The Q statistic and the I^2^ index were used to evaluate the heterogeneity across the studies.

**Results:**

Eight studies were ultimately included in this meta-analysis. In the overall analysis, no significant association was found between acne and prostate cancer risk (OR = 1.08, 95% CI 0.93–1.25). A significant heterogeneity was observed across studies (P = 0.006, I^2^ = 64.5%). In the subgroup analysis by study design, a significant association was observed in the cohort studies (OR = 1.51, 95% CI 1.19–1.93) but not in the case-control studies (OR = 0.98, 95% CI 0.86–1.12).

**Conclusions:**

In summary, this meta-analysis did not find an association between acne in adolescence and prostate cancer risk. However, because there was some heterogeneity in the overall analysis and a significant association was observed in the meta-analysis of the cohort studies, further well-designed large prospective studies are warranted to confirm our results.

## Introduction

Prostate cancer is the second most commonly diagnosed cancer in men worldwide, with 1.1 million new cases estimated to have occurred in 2012 [1]. Advanced age, African descent and a positive family history of prostate cancer are the only established risk factors for prostate cancer [2, 3]. Therefore, emerging studies are currently evaluating new markers that may help identify men with a high risk of prostate cancer, in whom the benefits of screening may outweigh the potential side effects [4, 5].

Recently, emerging studies have examined the association between acne and the risk of prostate cancer given that acne vulgaris is a proxy for androgen status [6]. In addition, *Propionibacterium acnes* (*P*. *acnes*), a skin bacterium closely associated with acne, is reported to be associated with prostatic inflammation and carcinogenesis [7–9]. Several observational studies have found a possible link between a history of acne and prostate cancer risk but with inconsistent results. A recent large prospective cohort study by Ugge et al. [10] indicated that acne was significantly associated with an increased risk for prostate cancer. The Glasgow Alumni Cohort Study suggested that individuals with acne in adolescence had a higher risk of adult prostate cancer mortality [11]. By contrast, the studies by Lightfoot et al. [12] and Cremers et al. [13] failed to establish a relationship between acne and prostate cancer risk. Given these controversial findings, we performed a systematic review and meta-analysis to summarize all relevant evidence. To the best of our knowledge, this is the first meta-analysis to assess the potential association between acne and prostate cancer risk.

## Materials and methods

### Literature search

A comprehensive literature search was performed in PubMed, Scopus, Web of Science, and Chinese National Knowledge Infrastructure (CNKI) databases up to March 2018 with the following search keywords: (acne or propionibacterium) and (prostatic neoplasms or prostatic cancer or prostate neoplasms or prostate cancer). We first evaluated the potentially relevant articles by checking their titles and abstracts. Any studies possibly matching the eligibility criteria were further evaluated by reading the full texts. We also examined the cited references lists from retrieved articles and reviews to identify any additional relevant studies. This systematic review and meta-analysis was planned, performed, and reported in adherence to the reporting and publication requirements of the Preferred Reporting Items for Systematic reviews and Meta-Analyses (PRISMA) statement [14].

### Study selection

Studies included in this meta-analysis met all of the following criteria: (i) the exposure of interest was acne; (ii) the outcome of interest was prostate cancer; (iii) the study had a cohort, nested case-control, or case-control design; and (iv) the study provided risk estimates with their corresponding 95% confidence intervals (CIs) or enough data to calculate them. There was no restriction on language or publication date.

### Study quality assessment

The method quality of each study was assessed by two independent reviewers (X.Z. and Y.L.) with the Newcastle-Ottawa Scale (NOS, http://www.ohri.ca/programs/clinical_epidemiology/oxford.asp). The NOS is an eight-item instrument that primarily assesses the following sections of each study: source of study population, study comparability, follow-up, and outcome of interest. We assigned NOS scores of <7 and ≥7 for low- and high-quality studies, respectively.

### Information extraction

Data were extracted by two independent reviewers (X.Z. and Y.L.) with a predefined information collection form. Any discrepancies were resolved by group consensus. We recorded the following data for each study: first author’s surname, the country in which the study was performed, publication year, study design, sample size, methods of exposure assessment, fully adjusted risk estimates with their 95% CIs, and adjusted confounders in the data analysis.

### Statistical methods

As prostate cancer is a rare disease, the relative risk (RR) and the hazard ratio (HR) were assumed to be approximately the same as the odds ratio (OR). The ORs and their 95% CIs were used as the study outcome to assess the strength of the relationship between acne and prostate cancer risk. Summary ORs with 95% CIs were estimated with a random effects model using the method of DerSimonian-Laird [15], which incorporates both within- and between-study variability. Subgroup analyses were performed according to study design, geographic region, study quality, number of cases, and publication year.

Heterogeneity across studies was evaluated using the Q statistic with a significance level set to P value < 0.10 [16]. I^2^ score (95% CI) was further used to assess the degree of heterogeneity (I^2^<25%, mild heterogeneity; I^2^ = 25–50%, moderate heterogeneity; I^2^>50%, large or extreme heterogeneity) [16]. Finally, a meta-regression and Galbraith plot analysis [17] were used to explore the possible sources of heterogeneity if any.

Sensitivity analysis was performed by repeating the pooled analysis after omission of each study in turn. A cumulative meta-analysis was performed by sorting each study by publication year. All of the statistical analyses were performed with Stata 10.0 (StataCorp, College Station, TX). A two-sided P value < 0.05 was considered statistically significant.

## Results

### Publication search and study characteristics

**Fig 1:**
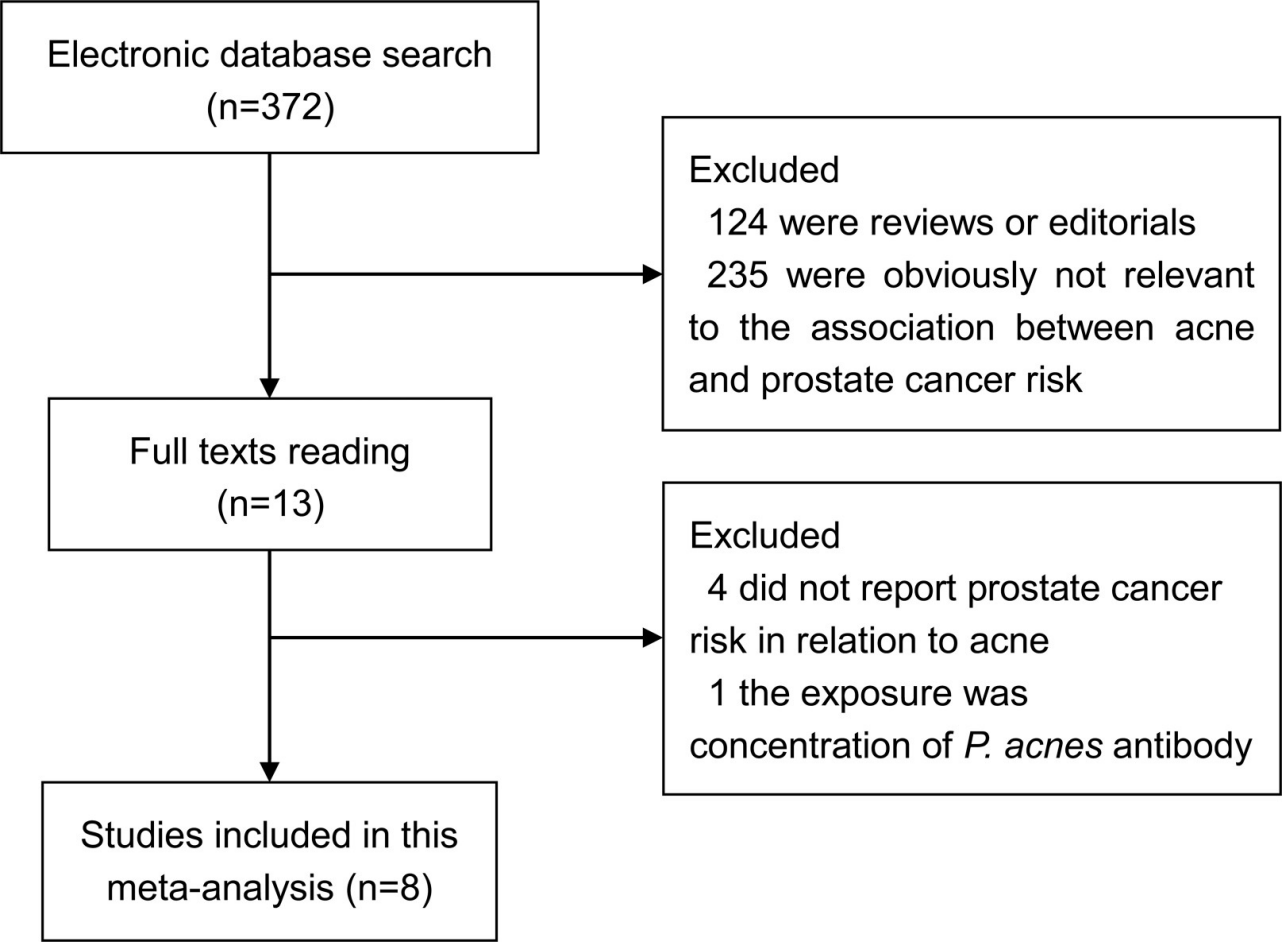
Literature search and selection process.

The detailed process of our literature search and selection is shown in Fig 1. A total of 372 studies were identified primarily from electronic databases. After excluding 124 reviews/editorials and 235 studies that were obviously not relevant (e.g., animal or cell line studies and studies concerning other types of cancer), 13 studies were evaluated by reading the full texts. Four studies that did not report prostate cancer risk in relation to acne and one study that reported the exposure as concentration of *P*. *acnes* antibody were also excluded. Eight studies [10–13, 18–21] were ultimately included in this meta-analysis to evaluate the association between acne and prostate cancer risk. These studies were published between 2003 and 2018 with a total of 10,145 cases. There were three cohort studies [10, 11, 20] and five case-control studies [12, 13, 18, 19, 21]. These studies were performed in the following geographic region: North America (n = 2), Europe (n = 4), and Oceania (n = 2). Information on acne was obtained by interview or self-administered questionnaire. The scores for study quality assessed by the NOS ranged from 5 to 8. The main characteristics of each study included in our meta-analysis are shown in Table 1.

**Table 1:** Study characteristics of published cohort and case-control studies on acne and the risk of prostate cancer.

Author, year of publication	Country and design	Cases/controls or cohort	Quality score	Acne assessment	Exposure comparison	OR/RR/HR (95% CI)	Matched or adjusted variables
Ugge et al., 2018	Sweden; Cohort	1,633/ 243,187	8	Interview	Acne versus no acne	1.43 (1.06–1.92)	Birth year, occupation, household crowding, height, BMI, physical capacity score, summary disease score, summary cognitive score, stress resilience score, erythrocyte sedimentation rate, erythrocyte volume fraction and residence
Nair-Shalliker et al., 2017	Australia; PCC	1,181/875	6	Questionnaire	Facial acne scarring versus no acne	0.88 (0.61–1.27)	Age
Rahman et al., 2015	UK; PCC	1,963/2,078	NA^a^	Questionnaire	Acne versus no acne	1.20 (1.04–1.40)	Age, family history of prostate cancer and ethnicity
Cremers et al., 2014	Netherlands; PCC	942/2,062	6	Questionnaire	Acne versus no acne	0.95 (0.80–1.12)	Age and family history of prostate cancer
Sutcliffe et al., 2007	USA; Cohort	2,147/34,629	7	Questionnaire	Tetracycline use ≥ 4 year duration versus none ^b^	1.70 (1.03–2.80)	Age, race/ethnicity and family history of prostate cancer
Galobardes et al., 2005	UK; Cohort	43/11,232 ^c^	6	Questionnaire	Acne versus no acne	1.67 (0.79–3.55)	Date of examination, father’s socioeconomic position, number of siblings, height, BMI, cigarette consumption, and systolic blood pressure.
Lightfoot et al., 2004	Canada; PCC	760/1,632	5	Questionnaire	Acne versus no acne	0.96 (0.79–1.17)	Age
Giles et al., 2003	Australia; PCC	1,476/1,409	7	Interview	Acne versus no acne	0.85 (0.70–1.04)	Age, study centre, calendar year, family history and country of birth

OR odds ratio, RR relative risk, HR hazard ratio, CI confidence interval, PCC population based case-control, BMI body mass index, NA not available.

a This study was published as conference abstract.

b Severe acne was measured by tetracycline use for 4-years or more.

c The outcome was prostate cancer mortality.

### Overall analysis and subgroup analysis

**Fig 2:**
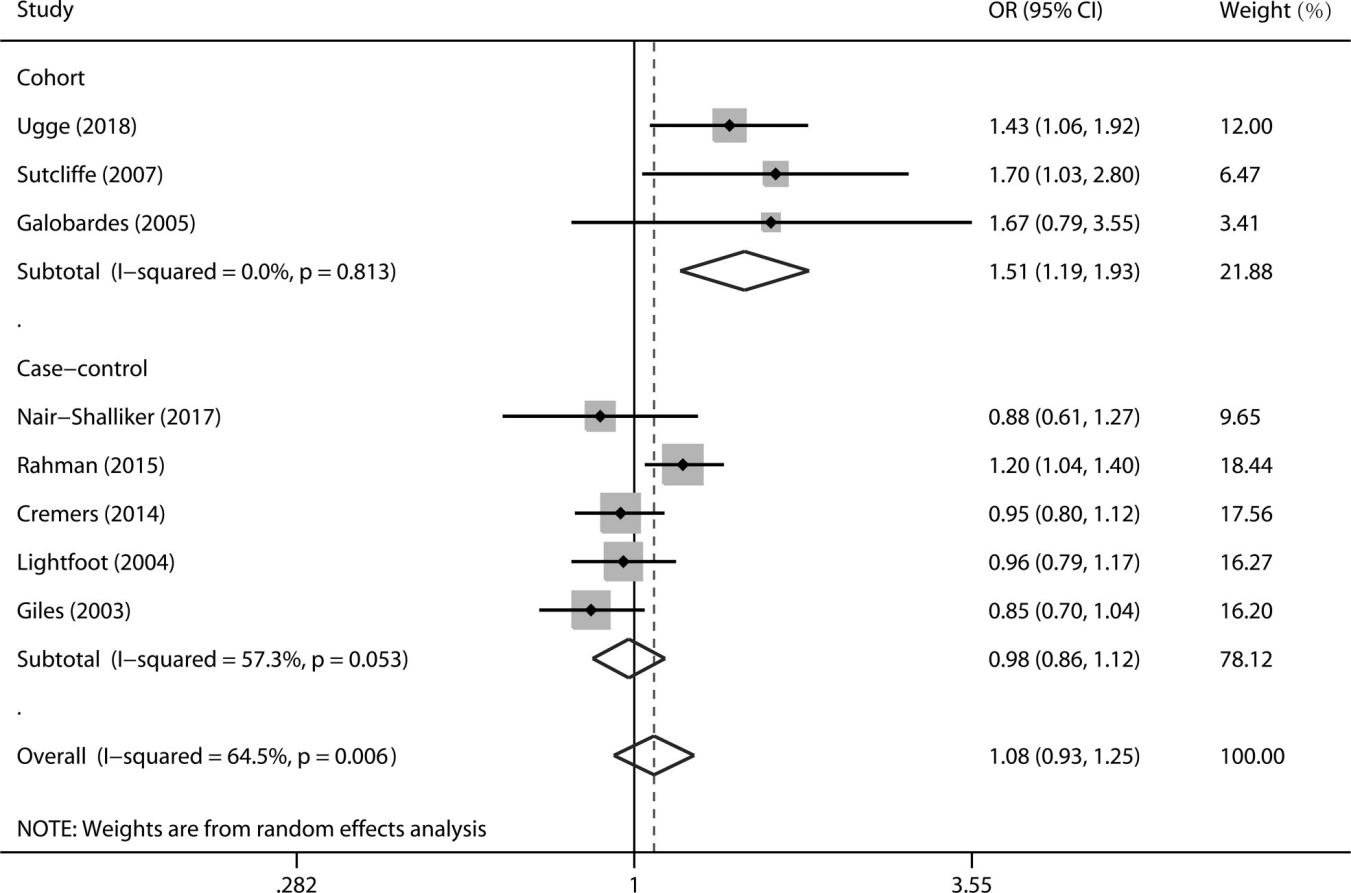
Forest plots showing risk estimates from cohort and case-control studies estimating the association between acne and the risk of prostate cancer.

The multivariable-adjusted ORs for each study and combination of all studies for acne versus no acne groups are shown in Fig 2. No significant association was found between acne and prostate cancer risk (OR = 1.08, 95% CI 0.93–1.25). Then, we performed subgroup analyses by study quality, study design, geographical region, number of cases, and publication year. In the subgroup analysis by study design, a significant association was observed in the cohort studies (OR = 1.51, 95% CI 1.19–1.93) but not in the case-control studies (OR = 0.98, 95% CI 0.86–1.12). No significant association was observed in any other subgroups (Table 2).

**Table 2:** Subgroup analyses for the relationship between acne and prostate cancer risk.

					Heterogeneity test		
Variables	Events	Individuals	OR (95% CI)	*P* for interaction	*Q*	*P*	*I*^*2*^ (95%CI, %)
**Total**	10,145	303,426	1.08 (0.93–1.25)		19.71	0.006	64.5 (24.1–83.4)
**Study design**				0.030			
Cohort	3,823	289,048	1.51 (1.19–1.93)		0.41	0.813	0.0 (0.0–89.6)
Case-control	6,322	14,378	0.98 (0.86–1.12)		9.36	0.053	57.3 (0.0–84.1)
**Study quality**				0.309			
High	5,299	29,1933	1.29 (0.87–1.91)		13.50	0.004	77.8 (39.8–91.8)
Low	2,883	7,452	0.95 (0.84–1.07)		0.17	0.917	0.0 (0.0–89.6)
**Geographical region**				0.146			
North America	2,907	37,021	1.22 (0.70–2.11)		4.35	0.037	77.0 (-)*
Europe	4,581	261,464	1.18 (0.96–1.44)		8.17	0.043	63.3 (0.0–87.6)
Oceania	2,657	4,941	0.86 (0.72–1.02)		0.03	0.870	0.0 (-)*
**No. of cases**				0.595			
> 1000	8,400	286,798	1.13 (0.90–1.42)		15.16	0.004	73.6 (34.3–89.4)
≤ 1000	1,745	16,628	0.97 (0.85–1.11)		2.08	0.354	3.8 (0.0–90.0)
**Publication year**				0.885			
> 2010	5,719	252,288	1.10 (0.91–1.33)		8.65	0.034	65.3 (0.0–88.2)
≤ 2010	4,426	51,138	1.07 (0.82–1.41)		8.55	0.036	64.9 (0.0–88.1)

OR odds ratio, CI confidence interval, No. number.

*Less than three studies were included in the subgroup, which was not eligible to calculate 95%CI.

### Evaluation of heterogeneity

In this meta-analysis, the Q statistic and the I^2^ index were used to evaluate the heterogeneity across the studies. Significant heterogeneity was observed among studies in the overall analysis (Fig 2, P = 0.006, I^2^ = 64.5%). Using Galbraith plot analysis, the study by Ugge et al. [10] was identified as the major source of heterogeneity (S1 Fig). Furthermore, we assessed heterogeneity across studies with a meta-regression method and study design was identified as a potential source of heterogeneity in the overall meta-analysis (Table 2, P for interaction = 0.030).

### Sensitivity analysis

**Fig 3:**
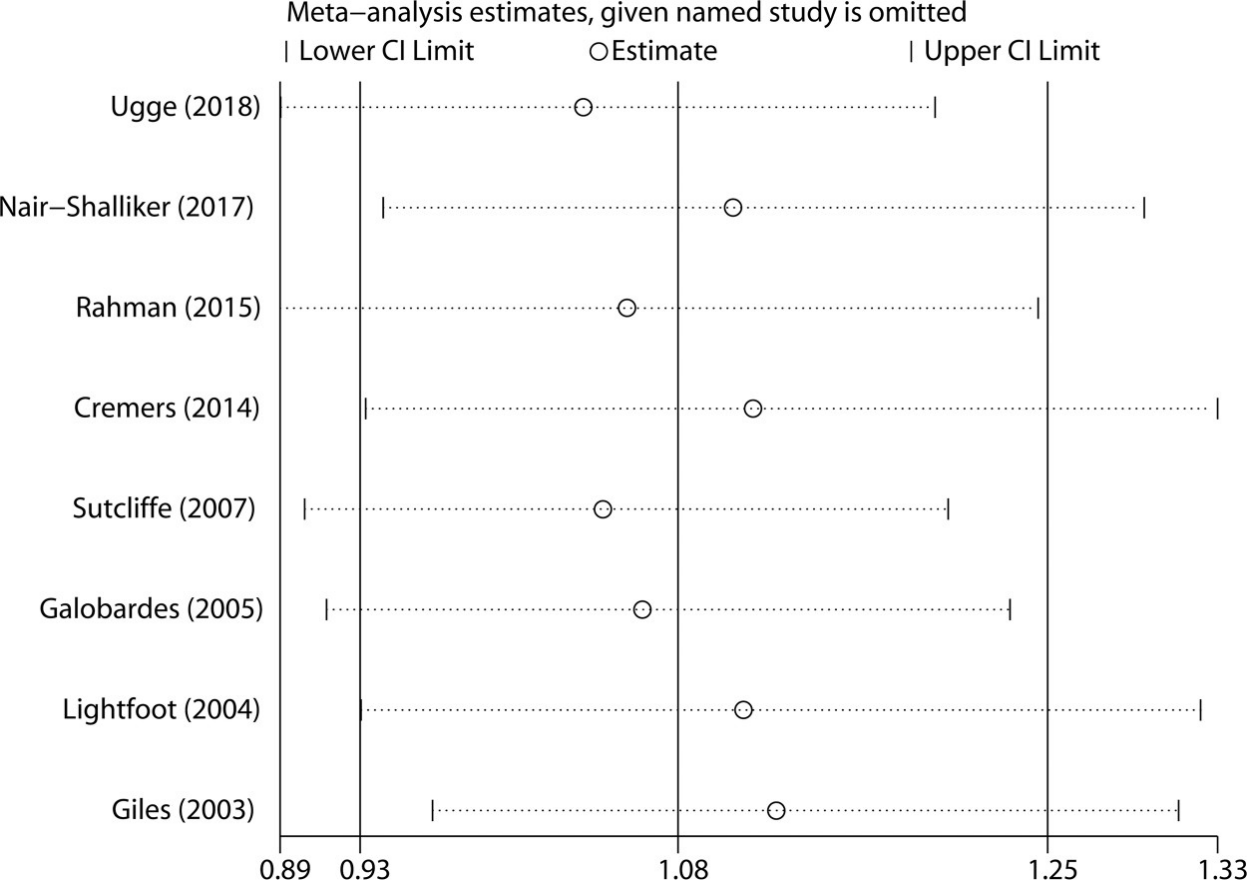
Sensitivity analysis was performed by omitting each study in turn and recalculating the pooled risk estimates.

A sensitivity analysis was performed to assess the influence of each study on the overall estimate. Meta-analysis estimates were computed by omitting one study at a time. The overall estimate (95% CIs) ranged from 1.03 (95% CI 0.89–1.20) to 1.12 (95% CI 0.96–1.31) after the omission of the study by Ugge et al.(2018) [10] and the study by Giles et al.(2003) [18], respectively (Fig 3).

### Cumulative meta-analysis

A cumulative meta-analysis was performed on the included studies sorted by publication year. As shown in S2 Fig, the association between acne and prostate cancer risk is presented in chronological order. The 95% CIs became narrower with cumulative sample size, indicating that the precision of risk estimates was progressively boosted by the continual addition of studies.

## Discussion

The present study summarized the current evidence of the relationship between acne in adolescence and prostate cancer risk using a meta-analysis of observational studies, including five case-control studies and three cohort studies. To the best of our knowledge, this is the first meta-analysis evaluating the association between acne and prostate cancer risk. The results indicated that acne was not significantly associated with the risk of prostate cancer (OR = 1.08, 95% CI 0.93–1.25).

When we assessed heterogeneity across the studies with a meta-regression method, we found that study design was a potential source of heterogeneity in the overall meta-analysis. The summary OR (95% CI) for the cohort studies was 1.51 (1.19–1.93) without obvious heterogeneity across the studies. However, no significant association was observed in the case-control studies, and this meta-analysis of 8 studies was outweighed by the case-control studies, which would tend to make the summary risk estimate skew toward the null. However, as cohort studies are generally superior to case-control studies, further large prospective cohort studies are warranted because of the positive findings in the subgroup of cohort studies.

This study had some strengths. A total of 10,145 prostate cancer cases were included in this meta-analysis; the large sample size provided reliable summary risk estimates. We extracted data from the most fully adjusted models in each study, which might minimize potential confounding factors. Various subgroup analyses, heterogeneity analyses, and sensitivity analyses were performed to evaluate the robustness of our findings.

The exact pathogenic mechanism of prostate cancer is still largely unknown. It has been hypothesized that chronic inflammation may contribute to prostate carcinogenesis [22]. *P*. *acnes* isolated from radical prostatectomy specimens have been reported to be positively related to the onset and extent of both acute and chronic prostate inflammation [23]. *P*. *acnes* were more common in men with prostate carcinoma than in the controls, and the cells infected with *P*. *acnes* had increased proliferation *in vivo* [8, 24, 25]. Therefore, *P*. *acnes* infection may be a contributing factor to the development of prostate cancer [26, 27]. In theory, incorporating some simple factors, such as age, family history, and new reported markers (e.g., HPV-16 infection [28] and hypertension [29]), into prostate cancer risk calculators may help identify men with a relatively high risk of prostate cancer. Thus, if there is a significant association between acne and prostate cancer, we may be able to reduce the risk of prostate cancer by clearing *P*. *acnes*. However, in this study, we failed to detect a potential link between a history of acne and prostate cancer risk.

This study had several limitations that should be acknowledged. First, substantial heterogeneity was detected across individual studies (P = 0.006, I^2^ = 64.5%), which might distort the pooled risk estimates. This is a large meta-analysis, and there is a direct link between meta-analysis size and detected heterogeneity. Thus, obvious heterogeneity is the norm, and it is great if heterogeneity has been identified and can be incorporated in the model [30]. Second, because only 8 studies were included in our meta-analysis, we were not able to perform publication bias tests or display a funnel plot. Third, the majority of the included studies adopted a case-control design, which may introduce selection bias and recall bias. Fourth, given the small number of studies included, the meta-regression and Cochran’s Q test may have limited power [31]. Finally, there were differences in how acne was defined. Some studies used severe acne as exposure, while other studies used any acne as exposure, which may contribute to the heterogeneity that was observed.

## Conclusions

In summary, this meta-analysis did not find an association between acne in adolescence and prostate cancer risk. Because of the obvious heterogeneity across the studies, especially heterogeneity by study design, and the significant association observed in a subgroup of cohort studies, further well-designed large prospective studies are warranted to confirm our preliminary findings.

## Supporting information

S1 Checklist PRISMA checklist. (DOC)

S1 Fig Results from a cumulative meta-analysis of the association between acne and prostate cancer risk. (TIF)

S2 Fig Funnel plot of acne and prostate cancer risk. (TIF)
