# Supplementary figures and images for: Is acne in adolescence associated with prostate cancer risk? Evidence from a meta-analysis
Source: PLoS One. 2018 Nov 7;13(11):e0206249. doi: 10.1371/journal.pone.0206249 (PMC6221330; doi:10.1371/journal.pone.0206249)

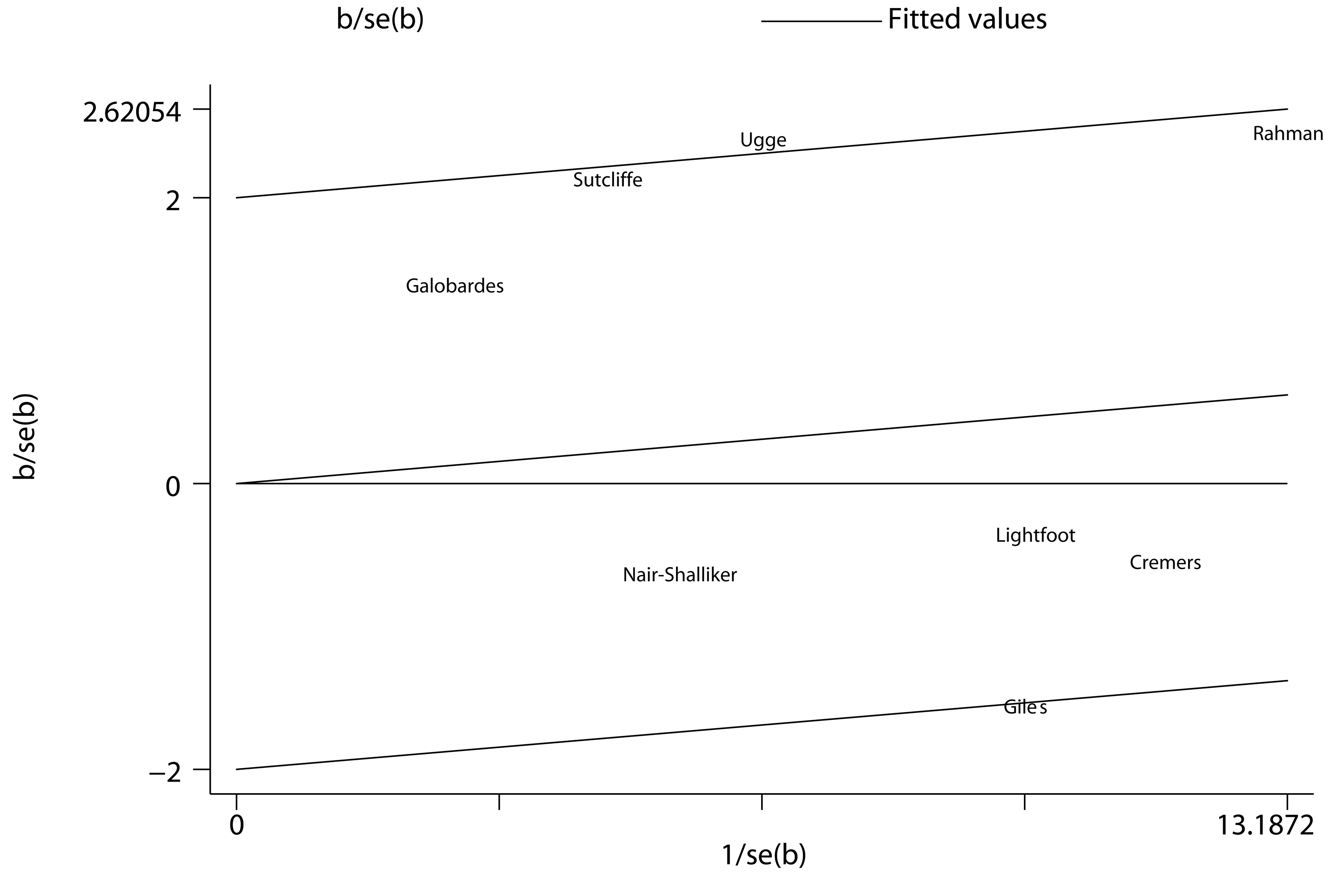

Supplement: S1 Fig — (TIF) [file pone.0206249.s002.tif]

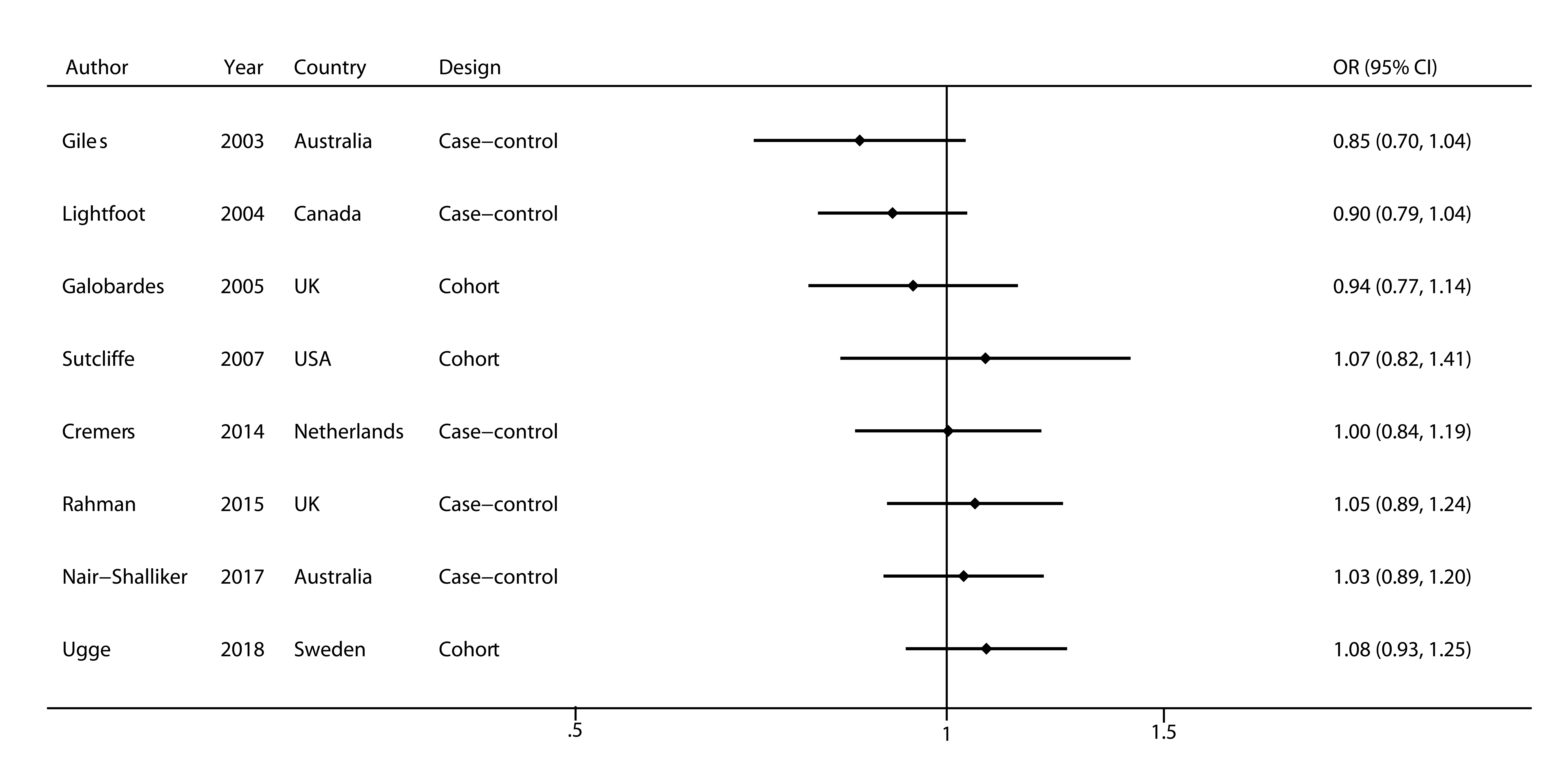

Supplement: S2 Fig — (TIF) [file pone.0206249.s003.tif]
